# Long-term effect of non-severe COVID-19 on pulmonary function, functional capacities and physical activities: a cross-section study in Sakaka Aljouf

**DOI:** 10.12688/f1000research.133516.1

**Published:** 2023-07-11

**Authors:** Maha Alshammari, ALSAYED SHANB, Mohammed Alsubaiei, Enas youssef

**Affiliations:** 1 Aljouf Cardiac Center, Ministry of Health, Sakaka Aljouf, Saudi Arabia; 2 Physical Therapy Department, College of Applied Medical Sciences, Imam Abdulrahman Bin Faisal University, Dammam, Eastern Province, Saudi Arabia; 3 Professor and chairman of Orthopedic Physical Therapy Department, Faculty of Physical Therapy, Cairo University, Giza, Egypt

**Keywords:** COVID-19, Pulmonary Function, Physical Activities, Functional Capacities

## Abstract

**Background:** COVID-19 has serious consequences on different body systems particularly the respiratory system with its impact on pulmonary function, functional capacities, physical activities, and personal performance. This study aimed to investigate the long-term effect of COVID-19 on pulmonary function, functional capacities, and physical activities in patients with non-severe COVID-19.

**Methods:** 160 individuals were selected to participate in a cross-section study. *Group-I:* 80 male and female patients with non-severe COVID-19 at least 3 months after the recovery time. *Group-*II:80 male and female matched participants. The spirometer, six-minute walk test (6MWT), and International Physical Activity Questionnaire (IPAQ) were used to assess pulmonary function, functional capacities, and physical activities respectively. Shapiro–Wilk’s test was used to test normality of data. The Mann–Whitney and independent t-tests were used to compare the significant differences between both groups.

**Results:** The results show significant differences in pulmonary function, functional capacities, and physical activities, between both COVID-19 and matched groups.

**Conclusion:** Pulmonary function, functional capacities, and physical activities are negatively influenced by COVID-19 as long-term consequences indicating the need for extended health care, and prescription of proper rehabilitative training programs for such patients whatever their severity degree of infection or history of hospitalization. Outcome reflections of the current results raise awareness for physical therapists to tailor the proper rehabilitative training programs for such patients.

## Introduction

Coronavirus disease 2019 (COVID-19) is caused by the severe acute respiratory syndrome coronavirus 2 (SARS-CoV-2), which began spreading on 31 December 2019 and had spread globally in the first months of 2020. ^1^ Although many patients with COVID-19 do not suffer from any symptoms and recover spontaneously without medical interventions, one in every six patients develops breathing difficulties and becomes seriously ill. ^1^ Until 29 September 2022, there had been 613,942,561 confirmed cases, including 6,520,263 deaths worldwide due to COVID-19. ^2^ Public health was forced to take specific protocols and precautions to prevent rapid spread of pandemic, and its associated economic crisis. ^3^ Tens of millions lost their jobs and increased poverty levels. ^4^ Consumption, investments, ^5^ work absenteeism, productivity, hospitality sectors and closures of factories all impacted negatively on income and supply. ^5^^,^^6^ COVID-19 affects people of all ages and seriously impacts different body systems. ^7^

Post-COVID-19 syndrome means the sequelae that develop during or after a SARS-CoV-2 infection and persist for more than 12 weeks. ^8^ It encompasses multi-organ sequelae beyond the acute phase of infection which ranges from physical and cognitive abnormalities to functional limitations, exercise impairments and deterioration of quality of life. ^9^^,^^10^ A massive number of humans suffered from multi-organ impairments resulting from wide distribution of angiotensin converting enzyme2 (ACE-2) receptors in extra-pulmonary tissues. ^11^^,^^12^ The pulmonary and cardiovascular systems are the most important impacted organs with their reflection on patient’s pulmonary function, functional capacities, physical activities, and quality of life. Similar coronavirus infection (SARS-CoV) caused impairments for two years after infection which are expected to occur for the survivors of COVID-19. ^13^ COVID-19 causes marked impairments in the diffusing lung capacity for carbon monoxide ( *D*LCO), total lung capacity, forced expiratory volume in one second and forced vital capacity ratio FEV _1_/FVC ratio, in addition, restrictions in small airways, ^14^^–^^16^ restrictive and obstructive patterns of the pulmonary function, ^15^ low quality of life ^15^^,^^16^ consolidation patterns with multifocal ground-glass opacities in computed tomography scans, ^17^ restrictions in both the 2MWT and FVC, ^17^ respiratory muscles dysfunction and lung fibrosis, ^18^^,^^19^ in addition to formation of pneumocytes. ^20^ Middle East respiratory syndrome (MERS) and severe acute respiratory syndrome (SARS) are the two previous viral infection outbreaks like the current COVID-19. ^20^ Abnormalities of the lung function are classified according to the American Thoracic Society as: normal, if both FVC and the FEV _1_/FVC ratio are in the normal range; obstructive pattern, if FEV _1_/FVC ratio is <70% of the normal predicted value and FEV _1_ <80% of the predicted; restrictive pattern, if FEV _1_/FVC ratio is ≥70% of the normal predicted value, and the total lung capacity <80% of the predicted value. If total lung capacity is not available, a reduction in the FVC <80% of predicted is considered as a restrictive pattern, small airway disease, if forced expiratory flow between 25% and 75% of FVC (FEF _25-75%_) is <65% of predicted value. ^21^ COVID-19 patients with cardiovascular and pulmonary comorbidities are more vulnerable to hospitalization, ^22^^,^^23^ and for developing neurological events, *e.g.*, acute cerebrovascular disease, conscious disturbance, and skeletal muscle injury. ^19^ Even though vaccination against COVID-19 can prevent hospitalization and severe infection. It has been adequate protection only against some long-COVID-19 symptoms, including cognitive dysfunction, sleeping disorders, and kidney diseases. ^24^

Even after recovery of survivors of COVID-19, almost 10-20% may suffer long-term consequences including fatigue, dyspnea, and impairments in both cognitive and daily functions. ^7^ Also, COVID-19 patients may be complicated with bladder dysfunction, severe urinary symptoms, ^25^^–^^27^ higher liver enzymes, ^28^ gastrointestinal symptoms, ^29^ psychotic disorders ^30^ and poly-neuromyopathy. ^31^ However, there were restrictions in the physical performance, physical activities, and impairments in sleep quality detected at 12 weeks post-COVID-19 infections. ^32^ Although time of walk improved significantly at the sixth month it still reduced on comparison with time of walk spent before COVID-19 for the same patients, ^33^ also, the physical activities and the one-minute standing test were impaired at discharge of patients with COVID-19. ^34^ Evidence of persistent physiological and radiographic changes is available in most patients who recovered from severe COVID-19, exercise capacity and dyspnea score improved over time to 12 months. ^35^ Patients with persistent dyspnea had several abnormalities during the 6MWT *e.g.*, greater restriction on spirometry, reduced functional capacity, and increased exertional symptoms. ^36^ As a result of variations in epidemiology and treatments for long-term sequels of COVID-19, it is considered as a new area of research. ^7^^,^^37^^,^^38^ There is need for more studies to investigate effects of COVID-19, particularly the long-term impact on the lungs and its reflections on the pulmonary function, physical activities, and functional capacities. The authors mainly concentrated on investigating hospitalized survivors and who experienced severe infection. ^19^^,^^39^^–^^41^ Non-severe COVID-19 survivors might be ignored during the pandemic so; further research was recommended particularly for those patients with mild and moderate degree of COVID-19 after recovery time. ^32^^,^^33^ Therefore, the current study aimed to investigate long-term effect of COVID-19 on pulmonary function, physical activities, and functional capacities in patients with non-severe degree after three months from recovery time in addition, evaluating time effect on the associated consequences.

## Methods

### Design of the study

A cross-section study.

### Sample size

The sample size was calculated by using an online tool ( http://www.stat.ubc.ca/~rollin/stats/ssize/n2a.html). It was based on the FEV _1_% (μ1 = 94.2, μ2 = 100.3, sigma = 13.1, and SD = 13.1 in the previous study. ^42^ The significant value is 0.05 with a power of.80.

***Ethical approval***

All procedures of the study were approved by the Ethics Research Committee of the Institutional Review Board of Imam Abdualrahman bin Faisal University (IRB-PGS-2021-03-427). Also, by the Research Ethics Committee in Qurayyat Health Affairs, Ministry of Health, Project no: 083, Saudi Arabia. This study was conducted in accordance with the Declaration of Helsinki at the out-patient clinic of the Physical Therapy Department of King Abdulaziz Specialist Hospital in Sakaka Aljouf, Ministry of Health- Saudi Arabia between September 2021 to June 2022. Prior to participation, all participants signed a consent form, and they were informed that the collected data would be submitted for publication.

*Subjects*

600 participants were screened from out-patient clinic of the Physical Therapy Department of King Abdulaziz Specialist Hospital in Sakaka Aljouf including *COVID-19 group:* 80 male and female patients (based on external examination of body characteristics) with confirmed non-severe COVID-19 at least 3 months from recovery time. Recovery is being free from fever and respiratory symptoms for at least 3 days followed by two negative polymerase chain reaction (PCR) tests 24 hours apart, or if PCR was not available, resolution of the clinical manifestations for 3 days and at least 10 days have passed from the appearance of the first symptom. ^43^ *Matched Group:* 80 male and female matched individuals (non-infected with COVID-19, their PCR was negative for COVID-19, no signs or symptoms of infection) who were invited to participate as control group.

*Inclusion criteria*

Male and female patients who diagnosed with non-severe COVID-19 at least after three months from recovery time and matched non-infected with COVID-19 participants, their age range from 25 to 55 years.

*Exclusion criteria*

All participants including non-infected individuals with COVID-19 and patients with COVID-19, acute infections, recent surgeries, unstable cardiovascular conditions, chronic respiratory diseases, neurological disease, mental illness, critically ill patients with intubation any other medical condition that contradict conduction of this research, who cannot walk, and smokers were excluded. ^44^^–^^46^

### Procedure of the study

Demographic data were recorded including weight, body mass index (BMI), oxygen saturation, heart rate, blood pressure, comorbidities, admission to the intensive care unit or hospitalization, severity degree of infection according to the classification of WHO progression scale. ^47^ This scale classifies severity of COVID-19 infection into five categories: 1-Uninfected with a 0 score, 2- Mild disease with a score ranging from 1-3, 3-Moderate disease with a score ranging from 4-5, 4-Severe disease with a score ranging from 6-9, and 5-Dead with score 10.

All participants underwent these outcome measures:

a) Pulmonary function was measured by using the Spirobank II spirometer (MIR www.spirometry.com). It is a validated device used for diagnosing and evaluating pulmonary diseases. ^48^ All participants underwent the test according to guidelines of the American Thoracic Society and European Respiratory Society (ATS/ERS). ^46^ The obtained parameters are FVC, FEV _1_, FEV _1_/FVC ratio, FEF _25-75%_, and peak expiratory flow (PEF). All measurements of pulmonary function testing (PFT) were expressed as absolute and percentage of predicted normal values (% predicted), the percentage of predicted normal values was calculated automatically based on age, sex, height, and ethnicity. ^49^ Each participant completed three accepted maneuvers and the highest value was recorded and used in the statistical analyses.
b) Physical activity was measured by using the International Physical Activity Questionnaire (IPAQ- Arabic version) which is valid and reliable. ^50^ It assesses physical activity during the last seven days throughout four domains: work-related physical activity, transportation-related physical activity, domestic and yard, and leisure time physical activity. Every participant was asked to answer each question in all domains. The scores are calculated for each domain and expressed as metabolic equivalent minutes per week (MET-minutes/week). The total physical activity score is calculated by summating the total scores for all domains, the physical activity score is classified into high, moderate, and low as 3000,600 and <300 MET-minutes/week respectively. ^51^
c) Functional capacity was measured by using the 6MWT: It is valid and reliable, and it has been approved to estimate sub-maximal exercise performance, daily physical activities, ^52^ physical endurance in older adults ^53^^–^^55^ and post-COVID-19 patients over 18 years. ^39^ Each participant was asked to walk independently on flat ground in 30-meters space for 6 minutes as fast as possible without oxygen inhalation, the results were expressed in meters. ^52^
d) Pulse oximeter is a valid and reliable device that was used to detect oxygen saturation and heart rate for every participant during 6MWT. ^56^
e) Modified Borg scale of dyspnea is a scale rated from 0 to 10. It was used to monitor severity of self-reported breathlessness during 6MWT. ^57^

### Statistical analysis

The collected data were analyzed using SPSS statistical software (version 25) and were tested for normality using Shapiro Wilk’s test. Group comparisons were done using independent t-test or Mann–Whitney test for data that had normal and abnormal distribution respectively. The Chi-squared test was used to compare the categorical variables. The COVID-19 group was divided into pre - and post 6-months sub-groups to determine time effect on the associated consequences whereas the Kruskal- Wallis’s test (Pairwise comparison) and one way ANOVA ( *post hoc* tests) were used to determine the significant differences among three groups. Statistical significance was set at P-value <0.05 with a confidence interval of 95%.

## Results

**Figure 1.**
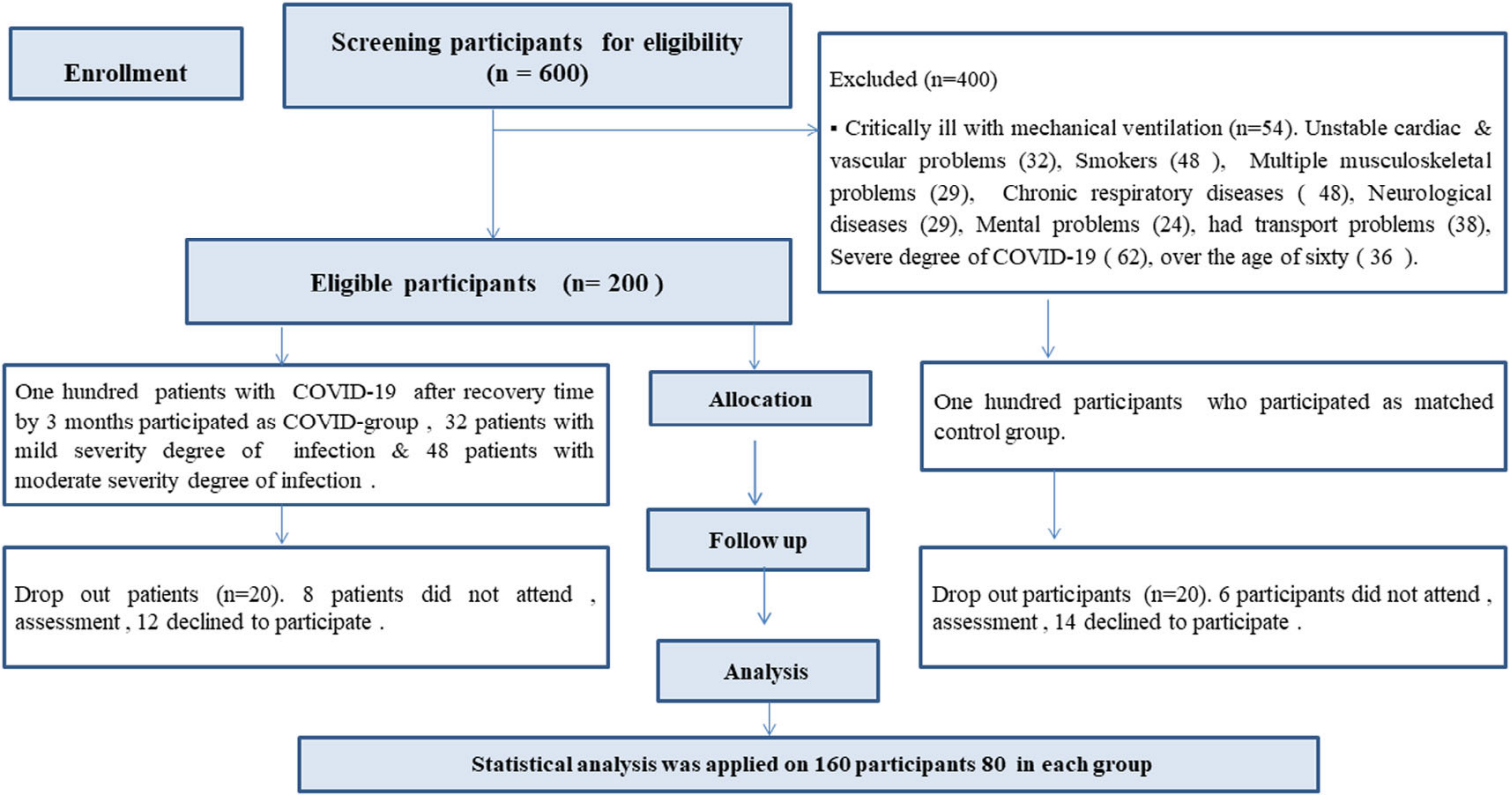
Flow chart of the participants' recruitment.

80 male and female patients with confirmed diagnosis of COVID-19 (COVID group), and another 80 matched participants non-infected with COVID-19 (Matched group) were recruited in this study ( Figure 1). Demographic and clinical characteristics of both groups including age, sex, BMI, and comorbidities were matched (P-value > 0.05), ( Table 1). There were significant differences in oxygen saturation between both groups (P-value = 0.003), 30 patients (37.5%) had comorbidities. The most common co-morbidities were obesity (31.25%), hypertension (2.5%) & diabetes (3.75%). Severity of infection was 32 patients (40%) with mild and 48 patients (60%) with moderate, 25 patients (31%) had restrictive pattern, and 13 participants (16%) in matched group, (P-value = 0.026), 17 patients (21%) with dyspnea & 48 patients (60%) were hospitalized ( Table 1).

**Table 1.** Demographic and clinical characteristics data of recruited participants.

Variables		COVID-19 group (Mean ± SD)	Matched group (Mean ± SD)	P-value
**Gender male & female N (%)**		M 25 (31.3%) F 55 (68.8%)	M 23 (28.7%) F 57 (71.3%)	0.730 ^†^ ^a^
**Age (in years)**		45.52 ± 9.61	44.24 ± 10.15	0.411 ^†^ ^b^
**Weight in kg**		71.04 ± 14.47	69.11 ± 13.73	0.389 ^†^ ^b^
**BMI (in kg/m** ^**2**^ **)**		26.45 ± 4.74	27.03 ± 6.03	0.496 ^†^ ^b^
**Comorbidities: N %**		30 (37.5%)	31 (38.75%)	0.965 ^†^ ^a^
**S**_**pO2**_		97.82 ± 0.72	98.15 ± 0.68	0.003 * ^b^
**Restrictive pattern N (%)**		25 (31%)	13 (16%)	0.026 * ^a^
**Overweight N (%)**		20 (25%)	17 (21.25%)	
**Obese N (%)**		25 (31.25%)	27 (33.75%)	
**HTN N (%)**		2 (2.5%)	2 (2.5%)	
**DM N (%)**		3 (3.75%)	2 (2.5%)	
**Time after recovery in months**		7.9 ± 3.12		
**Severity degree of infection N (%)**	**Mild**	32 (40%)		
	**Moderate**	48 (60%)		
**In-patients in ICU N (%)**		12 (15%)		
**Hospitalization N (%)**		48 (60%)		
**Dyspnea N (%)**		17 (21%)		
**Affected smell and taste N (%)**		8 (10%)		

BMI: body mass index, DM: diabetes mellitus, F: female, HTN: hypertension, M: male, S _pO2_: oxygen saturation.

^a^ Chi-squared.

^b^ Mann–Whitney test.

^†^ Non-significantly differences (P-value > 0.05).

* Significantly differences (P-value < 0.05).

After 3 months from time of recovery the results of pulmonary function tests show significant reductions in mean values of FVC%, FEV _1_%, FEV _1_/FVC Ratio%, FEF _25-75_%, and PEF% in COVID-19 group on comparison with matched group (P-value <0.05) ( Table 2). Also, mean values of distance of 6MWT and four domains of physical activity including work, transportation, domestic & yard, and leisure & free time reduced significantly in COVID-19 group on comparison with matched group (P-value < 0.05) ( Table 2).

**Table 2.** Mean values of PFT, 6MWT and IPAQ of COVID-19 and control groups.

Variables	COVID-19 group (Mean ± SD)	Matched group (Mean ± SD)	P-value
**FVC (Liters)**	3.21 ± 0.51	3.67 ± 0.90	<0.001 * ^b^
**FVC % pred (%)**	83.77 ± 8.53	90.95 ± 13.24	<0.001 * ^b^
**FEV**_**1**_ **(Liters)**	2.82 ± 0.48	3.16 ± 0.73	0.001 * ^c^
**FEV**_**1**_ **% pred (%)**	87.10 ± 9.52	95.46 ± 16.24	0.001 * ^b^
**FEV**_**1**_ **/FVC ratio (Liters)**	81.17 ± 7.89	84.39 ± 6.60	0.007 * ^b^
**FEV**_**1**_ **/FVC ratio % pred (%)**	99.66 ± 8.59	106.04 ± 10.82	0.001 * ^b^
**FEF**_**25-75%**_ **(Liters)**	3.34 ± 0.84	3.71 ± 0.99	0.013 * ^c^
**FEF**_**25-75%**_ **% pred (%)**	91.05 ± 19.59	101.34 ± 22.31	0.001 * ^b^
**PEF (Liters)**	6.05 ± 1.25	6.58 ± 1.24	0.008 * ^c^
**PEF % pred (%)**	97.29 ± 15.90	104.42 ± 16.92	0.011 * ^b^
**6MWT D**	377.51 ± 65.36	412.96 ± 47.88	<0.001 * ^c^
**PA of work**	1314 ± 1.89	2066 ± 2.21	0.005 * ^b^
**PA of transportations**	96.59 ± 156.02	211.21 ± 280.69	0.012 * ^b^
**PA of domestic and yard**	736.77 ± 680.2	1214.42 ± 1165.84	0.014 * ^b^
**PA of leisure and free time**	426.99 ± 465.59	1011.97 ± 1171.36	0.002 * ^b^

^b^ Mann Whitney test was used to determine significant differences in abnormal distributed variables.

^c^ Independent t-test was used to determine significant differences in normal distributed variables.

* Significantly difference P-value < 0.05. FEF _25-75%_ of pred: forced expiratory flows at 25-75% of FVC percentage of predicted, FEV _1_% of pred: Forced expiratory volume in the first second percentage of predicted, FEV _1_/FVC% of pred: forced expiratory volume in the first second and forced vital capacity ratio percentage of predicted, FVC% of pred: forced vital capacity percentage of predicted, PEF% of pred: peak expiratory flow percentage of predicted, 6MWT: Six minute walking test. IPAQ: international physical activities questionnaire, PA: physical activity.

The COVID-19 group was divided pre and post 6 months into two sub-groups to investigate the time effect on post-COVID-19 consequences. On comparison of pulmonary function and four domains of the IPAQ the results of the Kruskal-Wallis’ test show significant differences among three groups (P-value <0.05) except the predicted PEF% (P-value = 0.057) ( Table 3), also the results of one-way ANOVA test show significant differences in distance of the 6MWT among three groups (P-value = 0.01) ( Table 3).

**Table 3.** Mean values of PFT, 6MWT and IPAQ of COVID-subgroups and matched group.

Variables	Pre- 6-month (Mean ± SD)	Post- 6 month (Mean ± SD)	Matched group (Mean ± SD)	P-value
**FVC % pred**	82.93 ± 9.6	84.78 ± 8.59	90.71 ± 13.14	0.003 * ^d^
**FEV**_**1**_ **% pred**	87.73 ± 9.7	87.37 ± 10.48	95.15 ± 16.1	0.011 * ^e^
**FEV**_**1**_ **/FVC ratio % pred**	97.73 ± 9.12	101.2 ± 8.48	105.86 ± 10.77	0.005 * ^d^
**FEF**_**25-75%**_ **pred**	90.57 ± 18.59	92.0 ± 20. 48	101.04 ± 22.29	0.007 * ^e^
**PEF % pred (%)**	96.0 ± 18.37	98.49 ± 14.55	104.23 ± 16.93	0.057 ^†^ ^e^
**6MWT D**	383.97 ± 70.65	373.83 ± 61.78	413.33 ± 48.08	0.01 * ^e^
**PA of work**	1003 ± 1.61	1502 ± 2.02	2072 ± 2.22	0.010 * ^d^
**PA transportations**	96.43 ± 166.66	100.27 ± 151.69	210.35 ± 282.37	0.046 * ^d^
**PA domestic & yard**	838.8 ± 116.63	706.46 ± 98.92	1165.1 ± 131.08	0.014 * ^d^
**PA leisure & free time**	399.93 ± 497.6	475.31 ± 500.72	998.45 ± 1172.55	0.012 * ^d^

^d^ Kruskal Wallis test detects significant differences among three groups for abnormal distributed variables.

^e^ One way ANOVA tests significant differences among three groups for normal distributed variables.

* Significantly difference P-value < 0.05.

^†^ Non significantly difference P-value > 0.05.

The pairwise comparisons for pulmonary function and domains of the IPAQ in addition the *post hoc* tests for the 6MWT distance show significant differences on comparison of pre 6 months sub-group with matched group (P-value < 0.05) except the predicted PEF% (P-value = 0.057), and domestic & yard domain (P-value = 0.17) ( Table 4), while on comparison of the post 6 months sub-group with matched group the pairwise comparison and *post hoc* tests show significant differences in mean values of pulmonary function, 6MWT in addition only the both physical activity of domestic & yard and physical activity of leisure time domains of the IPAQ (P-value < 0.05) ( Table 5). In addition, there were non-significant differences between the pre-6 and post-6 months sub-group in all outcome measures (P-value > 0.05).

**Table 4.** Mean values of PFT, 6MWT and IPAQ of pre-6 month subgroup and matched group.

Variables	Pre- 6-month (Mean ± SD)	Matched group (Mean ± SD)	P-value
**FVC % pred**	82.93 ± 9.6	90.71 ± 13.14	0.002 * ^f^
**FEV**_**1**_ **% pred**	87.73 ± 9.7	95.15 ± 16.1	0.03 * ^f^
**FEV**_**1**_ **/FVC ratio % pred**	97.73 ± 9.12	105.86 ± 10.77	0.004 * ^f^
**FEF**_**25-75%**_ **pred**	90.57 ± 18.59	101.04 ± 22.29	0.019 * ^f^
**PEF % pred**	96.0 ± 18.37	104.23 ± 16.93	0.057 ^†^ ^f^
**6MWT D**	383.97 ± 70.65	413.33 ± 48.08	0.001 * ^g^
**PA of work**	1003 ± 1.61	2072 ± 2.22	0.004 * ^f^
**PA transportations**	96.43 ± 166.66	210.35 ± 282.37	0.024 * ^f^
**PA domestic & yard**	838.8 ± 116.63	1165.1 ± 131.08	0.17 ^†^ ^f^
**PA leisure& free time**	399.93 ± 497.6	998.45 ± 1172.55	0.008 * ^f^

^f^ The Pairwise comparison significant differences among three groups for abnormal distributed variables.

^g^ Post hoc tests showed significant differences among three groups for normal distributed variables.

* Significantly difference P-value < 0.05.

^†^ Non significantly difference P-value > 0.05.

**Table 5.** Mean values of PFT, 6MWT and IPAQ of post-6 month subgroup and matched group.

Variables	Post- 6 month (Mean ± SD)	Matched group (Mean ± SD)	P-value
**FVC % pred**	84.78 ± 8.59	90.71 ± 13.14	0.016 * ^f^
**FEV**_**1**_ **% pred**	87.37 ± 10.48	95.15 ± 16.1	0.007 * ^f^
**FEV**_**1**_ **/FVC ratio % pred**	101.2 ± 8.48	105.86 ± 10.77	0.022 * ^f^
**FEF**_**25-75%**_ **pred**	92.0 ± 20. 48	101.04 ± 22.29	0.006 * ^f^
**PEF % pred**	98.49 ± 14.55	104.23 ± 16.93	0.057 ^†^ ^f^
**6MWT D**	373.83 ± 61.78	413.33 ± 48.08	0.01 * ^g^
**PA of work**	1502 ± 2.02	2072 ± 2.22	0.072 ^†^ ^f^
**PA transportations**	100.27 ± 151.69	210.35 ± 282.37	0.088 ^†^ ^f^
**PA domestic & yard**	706.46 ± 98.92	1165.1 ± 131.08	0.004 * ^f^
**PA leisure & free time**	475.31 ± 500.72	998.45 ± 1172.55	0.032 * ^f^

^f^ The Pairwise comparison show significant differences among three groups for abnormal distributed variables.

^g^ Post hoc tests show significant differences significant differences among three groups for normal distributed variables.

* Significantly difference P-value < 0.05.

^†^ Non significantly difference P-value > 0.05.

## Discussion

COVID-19 is a new rapidly spreading epidemic, its initial symptoms may progress to long-term consequences. Results of the current study indicate that post-COVID-19 patients may experience chest abnormalities including reductions in pulmonary function, decreased functional capacity, and physical activities up to one year after recovery time. Sights of researchers were attracted to investigate them all over the world. Our findings agree with the results of Abdallah *et al*, Lorent *et al* & Salem *et al* they found significant reductions in mean values of FVC, FVC% predicted, FEV _1_, PEF, PEF% predicted at the third month of recovery on comparison with matched participants. ^40^^,^^58^^,^^59^ Restrictive pattern of impairments was observed in 50% of COVID-19 patients’ sample of Salem *et al.* ^40^ while it was 31% in the current study. A greater percent of restrictive pattern in findings of Salem *et al.* ^40^ may be due to their patients’ sample was COVID-19 patients with pneumonia or hospitalized (more complicated), whereas the current patients’ sample was selected with mild or moderate degree of infection. The current findings of pulmonary function are consistent with the findings of previous studies. Salem *et al* found significant reductions in pulmonary function of the survivors of COVID-19 after three months of discharge on comparison with matched controls. ^40^ Also, Abdallah *et al.* found reductions in the measured FVC, total lung capacity (TLC), and *D*LCO at the third month in hospitalized patients with severe COVID-19. ^58^ In contrast to our results the findings of Lerum *et al.* show normal pulmonary outcomes including lung function, 6MWT distance, oxygen saturation, dyspnea prevalence measured at the third month after hospital discharge. ^60^ Also, Eksombatchai *et al.* found non-significant differences in the pulmonary function of mild and moderate survivors COVID-19 with pneumonia. ^61^ The authors highlighted the absence of PFT data for their patients’ samples prior to occurrence of COVID-19. ^40^^,^^62^

The underlying mechanisms for COVID-19 multiple findings may be due to acute lung injury with diffuse alveolar damage which is associated with fibrotic changes and microthrombi in the pulmonary vasculature. ^63^ The restrictive impairment of the lung function may be caused by fibrotic changes in the lung and increase proinflammatory cytokines which recruit fibroblasts resulting in lung fibrosis. ^64^ The decline in pulmonary function results from the respiratory muscles fatigue as a significant improvement of PFT after pulmonary rehabilitation for COVID-19 survivors, ^65^ the results of PFT are also influenced by several factors *e.g.*, sex and body type. ^66^

Our findings show significant reductions in measured parameters of pulmonary function, 6MWT distance and domains of physical activities in patients with COVID-19 after 3 months, pre 6 months and post 6 months on comparison with the matched group. There are progressive improvements on comparison of pre with post 6 months as a time effect and being non-significant may be due to patients’ sample of pre 6 months was not the same patients’ sample of post 6 months. The current results are consistent with findings of Magdy *et al.* determined lower limits in lung function (<80%) and non-statistically significant differences in the pulmonary function at 3 and 6 months post-infection. ^67^ whereas existing significant improvements at one year follow-up. ^59^ This finding does not contradict with our results as they compared the same patients at 3 months, 6 months and after one year not the case in the current study where the patients’ sample was compared at 3 months, pre 6 months and after 6 months to matched control. Also, Zhang *et al.* found 20% of the survivors of COVID-19 had FEV _1_/FVC below 70% of predicted values at the eighth month. ^62^

The current results contradict the findings of Wu *et al.* as they found significant increases in pulmonary function at 3, 6, 9 and 12-month interval measures post-infection (time effect) ^35^ this may be also due to the authors did interval assessments for the same COVID-19 patients. They found high rate of dyspneic patients (81%) measured at the third month whereas it was 21% in the current study. This may be their sample included only severe COVID-19 patients whereas the sample in the current study included both mild and moderate degree of COVID-19. Also, Madrid-Mejía *et al.* determined improvements in PFTs at the sixth month of infection compared to the results of the same participants at the third month after infection. ^68^ The variations in the time of evaluation in different studies may explain the differences in the results. ^15^^,^^40^^,^^69^

In our study, despite the result of the functional capacities (6MWT distance) show significant reductions in the COVID-19 group after 3 months, pre and post 6 months from recovery on comparison with the matched control, there are non-significant increases at both pre and post 6 months. This finding is consistent with the results of Magdy *et al.* who determined significant reductions in the 6MWT results of the survivors of COVID-19 on comparison with the normative data, ^40^ whereas a significant improvement was determined in the 6MWT at the sixth month in regarding the third-month follow-up. ^40^ They referred their findings to the extended period of hospital stay and extra usage of corticosteroids which could influence the muscles resulting in muscle wasting and myopathy. ^40^^,^^70^^,^^71^ also, Calabrese *et al.* demonstrated significant reductions in the FVC %, *D*LCO, low oxygen saturation ( *S*_pO2_) (>90%) during the 6MWT with higher dyspnea. ^41^ In addition, Raman *et al.* found significant reductions in the distance of the 6MWT for COVID-19 patients on comparison with controls. ^72^ They referred this limited exercise capacity to muscle wasting that caused by the catabolic state resulting from severe illness, and potentially inflammation. ^72^^–^^74^ While Abdallah *et al.* reflected the persistence of breathlessness and limitation in exercise capacity at the third month to the residual defects in TLC. ^58^ In addition, the recovery of the physical function within the first 6 months of patients after SARS-COV was incomplete as it lasts for one to two years. ^13^ In addition, Magdy *et al.* found significant increases in the 6MWT distance at the 6-month follow-up. ^67^ Accordingly, the lower results of the 6MWT distance may be attributed to the higher BMI, and high number of female participants in the current study. The distance of the 6MWT is negatively influenced by sex and body type. ^52^ On contrary to the current findings Wu *et al* determined significant improvements in the 6MWT at 3-, 6-, 9-, and 12-month interval measures post-infection. ^35^

Our results agree with the findings of Belli *et al*. they found patients with COVID-19 suffer from impairments in physical functions and fitness, as 33.3% of patients had impaired physical fitness, and 17.5% with moderate scores in activities of daily livings performance. ^75^ Cao *et al*. also stated that performance in the 6MWT was significantly lower in post COVID-19 patients than in health controls. ^76^ Lower performance in the 6MWT was reported in patients with severe/critical COVID-19 compared to patients with mild/moderate disease at baseline. ^77^ On contrary to the results of the current study the findings of Lerum *et al.* they concluded non-significant differences in the results of the 6MWT between ICU and non-ICU groups and Eksombatchai *et al.* they found statistically typical results for the 6MWT among three groups, while the severe infection group showed lower results when compared with the mild and moderate infection groups but not statistically significant. ^60^^,^^61^ The results of oxygen saturation show non- significant differences (p = 0.201) among groups for both pre and post 6MWT. They referred the reductions in their results to the higher BMI and older age in the severe group.

In the current study, the results of the IPAQ significantly reduced in all domains in the COVID-19 survivors’ group on comparison with the matched group after 3 months. The current findings are supported with the results of Tanriverdi *et al.* found poor physical activities and impaired hand grip power at least three months of survivors of COVID-19. ^32^ Paneroni *et al.* who determined impaired physical activities at the discharge time. ^34^ As a result of improvements in physical activities with time effects on comparing survivors of COVID-19 of pre and post 6 months with the matched controls, significant differences were determined in some IPAQ domains. Also, the current findings supported the findings of Delbressine *et al.* who found significant improvements in physical activities in the survivors of COVID-19 at the sixth month compared with results at the third month post-infection. ^33^ In the current study, the IPAQ questionnaire was used to assess the physical activities during the last seven days only, which may not give an accurate perception of the physical activities as it could be affected by other factors *e.g.*, the work domain could be lower for some participants because they were on vacation for the last seven days. The transportation domain may be lower for some patients because they must use vehicles due to the hot weather. Some participants did not have gardens or backyards, which reduces their domestic and yard scores. Other participants may have lower leisure time domain scores because they did not feel well to walk or did exercise during the last seven days in convalescent stage. Although results of outcome measures show significant reductions in pulmonary function, physical activities, and functional capacity after 3 months, pre 6 months and post 6 months on comparison to matched participants there are general progressive improvements as time effects but in comparison with matched control still need extended health care and prescribed proper rehabilitative training programs whatever severity of infection or history of hospitalization.

### Limitations

There was a lack of data on health conditions of patients prior to contracting COVID-19 so the authors tried to overcome this limitation by including a matched control group, small sample size, usage of simple spirometry approach, lack of *D*LCO and plethysmography. Despite these limitations, the authors believe that the results of this study contribute to filling a significant knowledge gap about consequences of COVID-19 after 3 months of recovery time.

### Recommendations

Further studies to investigate effectiveness of COVID-19 long-term complications and follow-up for patients with different severity of infections and effectiveness of individualized comprehensive rehabilitative programs for such patients.

## Conclusion

Pulmonary function, functional capacities, and physical activities are negatively influenced by COVID-19 as long-term consequences indicating the need for extended health care, and proper rehabilitative training programs for those patients whatever the severity degree of infection or history of hospitalization. Gaining the deepest knowledge that enables physical therapists to tailor the appropriate rehabilitative training program for such patients.

## Data Availability

Figshare: Sensory perception, https://doi.org/10.6084/m9.figshare.23153540.v1. ^78^ This project contains the following underlying data:
- BASE DE DATOS EVALUACIÓN SENSORIAL 24 05 2023.xls (Data for tastings carried out with students. The samples were 4 cereal bars made with cereal grains and with different percentages of ant flour.) Data are available under the terms of the Creative Commons Attribution 4.0 International license (CC-BY 4.0).
