# Supplementary material for: Long-term effect of non-severe COVID-19 on pulmonary function, exercise capacities and physical activities: a cross-section study in Sakaka Aljouf
Source: F1000Res. 2024 Mar 4;12:809. Originally published 2023 Jul 11. [Version 5] doi: 10.12688/f1000research.133516.5 (PMC10973945; doi:10.12688/f1000research.133516.5)
Supplement: Peer review history

# Reviewer report: Reviewer response for version 5

**DOI:** 10.5256/f1000research.163263.r252539

**Published:** 2024-03-26

**Authors:** Asir John Samuel, Vencita Priyanka Aranha

**Affiliations:** 1 Yenepoya Physiotheapy College, Mangalore, Karnataka, India; 2 Physiotherapy, Aikya Physiotherapy and Rehabilitation, Karkala, Karnataka, India; 3 Aikya Physiotherapy and Rehabilitation Centre, Karkala, Karnataka, India

We agree that the writing style of the interquartile range (IQR) in table 1 and table 2 is correct. However, when they are combined with median, the syntax of using median & IQR is incorrect. It should be re-written as, Median (25th quartile, 75th quartile) instead of median +/-  (25th quartile, 75th quartile). "+/-" to be removed. Otherwise fine. Can be considered Approved.

Is the work clearly and accurately presented and does it cite the current literature?

Partly

If applicable, is the statistical analysis and its interpretation appropriate?

No

Are all the source data underlying the results available to ensure full reproducibility?

No

Is the study design appropriate and is the work technically sound?

Partly

Are the conclusions drawn adequately supported by the results?

Yes

Are sufficient details of methods and analysis provided to allow replication by others?

Partly

Reviewer Expertise:

Physical activity, pulmonary function, COVID-19 impact

We confirm that we have read this submission and believe that we have an appropriate level of expertise to confirm that it is of an acceptable scientific standard.

# Reviewer report: Reviewer response for version 5

**DOI:** 10.5256/f1000research.163263.r252538

**Published:** 2024-03-20

**Authors:** Ashraf Abdelaal Mohamed Abdelaal

**Affiliations:** 1 Department of Physiotherapy, Faculty of Applied Medical Sciences, Umm Al-Qura University, Makkah, Saudi Arabia

No further comments.

Is the work clearly and accurately presented and does it cite the current literature?

Yes

If applicable, is the statistical analysis and its interpretation appropriate?

Partly

Are all the source data underlying the results available to ensure full reproducibility?

Yes

Is the study design appropriate and is the work technically sound?

Yes

Are the conclusions drawn adequately supported by the results?

Yes

Are sufficient details of methods and analysis provided to allow replication by others?

Yes

Reviewer Expertise:

Physiotherapy for cardiovascular/pulmonary disorders and geriatrics, cardiopulmonary rehabilitation.

I confirm that I have read this submission and believe that I have an appropriate level of expertise to confirm that it is of an acceptable scientific standard.

# Reviewer report: Reviewer response for version 4

**DOI:** 10.5256/f1000research.161031.r236426

**Published:** 2024-02-28

**Authors:** Asir John Samuel, Vencita Priyanka Aranha

**Affiliations:** 1 Yenepoya Physiotheapy College, Mangalore, Karnataka, India; 2 Physiotherapy, Aikya Physiotherapy and Rehabilitation, Karkala, Karnataka, India; 3 Aikya Physiotherapy and Rehabilitation Centre, Karkala, Karnataka, India

**Reply to the response of the first comment:**

 "maximizing the sample size is healthy" is unjustifiable. Increasing sample size may result in considering small differences to be statistically significant differences, even though they are clinically insignificant.

**Reply to the response of the second comment:**

 Satisfied with the second response

**Reply to the response of the third comment:**

 Though means & standard deviation in all tables was replaced with median & IQR, the syntax of using median & IQR is incorrect. It should be re-written as, Median (25th quartile, 75th quartile). Moreover, table 2 still has the term, 'Mean values', even though they were represented as median & IQR. Satisfied with other responses for the third comment.

Is the work clearly and accurately presented and does it cite the current literature?

Partly

If applicable, is the statistical analysis and its interpretation appropriate?

No

Are all the source data underlying the results available to ensure full reproducibility?

No

Is the study design appropriate and is the work technically sound?

Partly

Are the conclusions drawn adequately supported by the results?

Yes

Are sufficient details of methods and analysis provided to allow replication by others?

Partly

Reviewer Expertise:

Physical activity, pulmonary function, COVID-19 impact

We confirm that we have read this submission and believe that we have an appropriate level of expertise to confirm that it is of an acceptable scientific standard, however we have significant reservations, as outlined above.

# Reviewer report: Reviewer response for version 4

**DOI:** 10.5256/f1000research.161031.r236427

**Published:** 2024-01-29

**Authors:** Ashraf Abdelaal Mohamed Abdelaal

**Affiliations:** 1 Department of Physiotherapy, Faculty of Applied Medical Sciences, Umm Al-Qura University, Makkah, Saudi Arabia

Authors justified the suggested corrections

Is the work clearly and accurately presented and does it cite the current literature?

Yes

If applicable, is the statistical analysis and its interpretation appropriate?

Partly

Are all the source data underlying the results available to ensure full reproducibility?

Yes

Is the study design appropriate and is the work technically sound?

Yes

Are the conclusions drawn adequately supported by the results?

Yes

Are sufficient details of methods and analysis provided to allow replication by others?

Yes

Reviewer Expertise:

Physiotherapy for cardiovascular/pulmonary disorders and geriatrics, cardiopulmonary rehabilitation.

I confirm that I have read this submission and believe that I have an appropriate level of expertise to confirm that it is of an acceptable scientific standard.

# Reviewer report: Reviewer response for version 3

**DOI:** 10.5256/f1000research.159022.r227200

**Published:** 2023-12-19

**Authors:** Asir John Samuel, Vencita Priyanka Aranha

**Affiliations:** 1 Yenepoya Physiotheapy College, Mangalore, Karnataka, India; 2 Physiotherapy, Aikya Physiotherapy and Rehabilitation, Karkala, Karnataka, India; 3 Aikya Physiotherapy and Rehabilitation Centre, Karkala, Karnataka, India

**Methods:**

*Sample size:*

- By calculation, it was estimated to be n=73 in each group. Then, why n=7 samples were added to each group? It is unethical to utilize 14 human valuable times when the same could have arrived without including them. Please justify.

Procedure of the study:

- c) Functional capacity was measured by using the 6MWT: "without oxygen inhalation" is not correct. It should be changed to, "without oxygen support or assisted ventilation".

**Results:**

- Figure 1 – Still Not correct. In a cross-sectional study, how is dropout possible? Please remove the word, “Dropout” from, Figure 1
- In Table 1 to Table 4, if the data does not follow normal distribution, then it should be expressed as a median with interquartile range (IQR) or geometric mean with 95% confidence interval. Mean with SD is not appropriate. Modify accordingly. Even expressing in 95% confidence interval is not appropriate. Moreover, the way of representing 95% confidence interval is not correct. Table 4 is not necessary. Why have to express it in Mean with SD which is not appropriate?

Is the work clearly and accurately presented and does it cite the current literature?

Partly

If applicable, is the statistical analysis and its interpretation appropriate?

No

Are all the source data underlying the results available to ensure full reproducibility?

No

Is the study design appropriate and is the work technically sound?

Partly

Are the conclusions drawn adequately supported by the results?

Yes

Are sufficient details of methods and analysis provided to allow replication by others?

Partly

Reviewer Expertise:

Physical activity, pulmonary function, COVID-19 impact

We confirm that we have read this submission and believe that we have an appropriate level of expertise to confirm that it is of an acceptable scientific standard, however we have significant reservations, as outlined above.

# Response

**Published:** 2024-01-01

**Authors:** ALSAYED SHANB

**Affiliations:** Physical Therapy, Imam Abdulrahman Bin Faisal University, Dammam, Eastern Province, Saudi Arabia

Dear respected reviewer  Thanks for your valuable and important comments.

**First comment**

 Sample size: By calculation, it was estimated to be n=73 in each group. Then, why n=7 samples were added to each group? It is unethical to utilize 14 human valuable times when the same could have arrived without including them. Please justify.

**Response**

**1- **The sample size is considered the minimum number of participants must be included till the results analysis and statistical outputs to be valid and reliable, so the extra numbers make our results more valid and reliable.

 2-The calculated sample size   gives us an estimation of the minimum sample size,so  maximizing the sample size is healthy. Sample sizes are calculated to detect the smallest clinically significant difference. to detect the smallest clinically significant difference, it has more than adequate power to detect bigger differences. https://www.ndi.org/sites/default/files/samplesizecalculation.pdf page 17

*3-Overestimating *the same size could be done to minimize the effects of a few participants' incomplete data which is removed from analysis. 

**Second comment**

 Functional capacity was measured by using the 6MWT: "without oxygen inhalation" is not correct. It should be changed to, "without oxygen support or assisted ventilation".

**Response**

 The statement is rephrased to

  Functional capacity was measured by using the 6MWT without oxygen support.

**Third comment**

 Figure 1 – Still Not correct. In a cross-sectional study, how is dropout possible? Please remove the word, “Dropout” from, Figure 1

 In Table 1 to Table 4, if the data does not follow normal distribution, then it should be expressed as a median with interquartile range (IQR) or geometric mean with 95% confidence interval. Mean with SD is not appropriate. Modify accordingly. Even expressing in 95% confidence interval is not appropriate. Moreover, the way of representing 95% confidence interval is not correct. Table 4 is not necessary. Why have to express it in Mean with SD which is not appropriate?

**Response**

**a-The figure was updated and the word drop out was removed**

**b- the means & standard deviation in all table was replaced with median & IQR**

**c- Table 4 as you stated it was canceled and the statements were rephrased**

**d- the writing  style of confidence interval was changed**

**very thanks for your comments**

# Reviewer report: Reviewer response for version 2

**DOI:** 10.5256/f1000research.155029.r210129

**Published:** 2023-10-31

**Authors:** Asir John Samuel, Vencita Priyanka Aranha

**Affiliations:** 1 Yenepoya Physiotheapy College, Mangalore, Karnataka, India; 2 Physiotherapy, Aikya Physiotherapy and Rehabilitation, Karkala, Karnataka, India; 3 Aikya Physiotherapy and Rehabilitation Centre, Karkala, Karnataka, India

**Abstract:**

Background:

- Retain either, "physical activities" or "physical performance".
- Avoid duplicating similar words, "pulmonary function, functional capacities, physical activities" in the same paragraph.

Methods:

- " *Group-*II *:*80 male and female matched participants" - Matched? (matched non-infected with COVID-19) participants. Non-COVID-19 or ? to be added.
- As the sample size is >50, Shapiro–Wilk’s test will not be appropriate to test the normality of data. Kolmogorov–Smirnov test would be more appropriate. 

Results:

- Add appropriate "p-value" ie, 0.05 or 0.01 or?

**Introduction:**

- Overall, the introduction is lengthy. Generally, the introduction should be 10% to a maximum of 20% of the total manuscript length.
- Introduction should be drafted in 3 paragraphs. Please refer to this article for detailed description: Armağan (2013 ^1^).
- 1st Paragraph – Provide information about the general topic of the article in the light of the current literature which paves the way for the disclosure of the objective of the manuscript (what is known?).
- 2nd Paragraph – Focus on the specific subject matter, and the issue to be focused on should be dealt with, the problem should be brought forth, and fundamental references related to the topic should be discussed (what is unknown?).
- 3rd Paragraph - Finally, our recommendations for solution should be described, in other words our aim should be communicated. When these steps are followed in that order, the reader can track the problem, and its solution from his/her own perspective under the light of current literature (what are you going to do?).

**Methods:**

*Sample size:*

- Change, "sigma = 13.1, and SD = 13.1" as "sigma/SD = 13.1" as both denote the same meaning.
- By calculation, it was estimated to be n=73 in each group. Then, why n=7 samples were added to each group? It is unethical to utilize 14 human valuable times when the same could have arrived without including them. Please justify.

*Subjects:*

- (based on external examination of body characteristics) - reference or details to be added.

*Inclusion criteria:*

- Male and female patients who diagnosed with **mild & moderate** COVID-19 - Define "mild COVID-19" and "moderate COVID-19" or add the reference for the same.

*Exclusion criteria:*

- Patients with **sever** COVID-19 - "severe" spelling mistake. Define "severe COVID-19" or add the reference for the same.

Procedure of the study:

- c) Functional capacity was measured by using the 6MWT: "Each participant was asked to walk independently on flat ground" - describe the nature of the flat ground like, non-slippery, well illuminated, etc, and walk with footwear or without footwear. 
- d) Pulse oximeter - how was the pulse oximeter held - fastened to the fingers or any other part while walking as fast as possible? Add the practical issues that might help in replicating the study in the future. 

Statistical analysis:

- Shapiro Wilk’s test - to be replaced with Kolmogorov–Smirnov test. As the sample size is >50, Shapiro–Wilk’s test will not be appropriate to test the normality of data. Kolmogorov–Smirnov test would be more appropriate. 
- Avoid using abnormal distribution. Use, not normal distribution.
- Kruskal-Wallis’s test  - When two groups (Group-I) and (Group-II), why was Kruskal-Wallis’s test used? Justify. Pre 6 months and post 6 months descriptions are not clear. If COVID-19 group participants were divided, then each group would not have an appropriate sample size to detect the statistical difference and statistical comments could not be made. Add more clarity. 

**Results:**

- Figure 1 - Not correct. In a cross-sectional study, how is dropout possible? Please justify.
- In Table 1 to Table 5, if the data does not follow normal distribution, then it should be expressed as a median with interquartile range (IQR) or geometric mean with 95% confidence interval. Mean with SD is not appropriate. Modify accordingly.
- Moreover, Table 4 and Table 5 have duplicated values which were already presented in Table 3. Avoid this duplication. Tables 3-5 could be merged.

**Discussion:**

- Add strengths of the study.

**Conclusion:**

- Conclude regarding, non-severe COVID-19 and Sakaka Aljouf. These are two key facts to be added.

Is the work clearly and accurately presented and does it cite the current literature?

Partly

If applicable, is the statistical analysis and its interpretation appropriate?

No

Are all the source data underlying the results available to ensure full reproducibility?

No

Is the study design appropriate and is the work technically sound?

Partly

Are the conclusions drawn adequately supported by the results?

Yes

Are sufficient details of methods and analysis provided to allow replication by others?

Partly

Reviewer Expertise:

Physical activity, pulmonary function, COVID-19 impact

We confirm that we have read this submission and believe that we have an appropriate level of expertise to confirm that it is of an acceptable scientific standard, however we have significant reservations, as outlined above.

## References

[1] : How to write an introduction section of a scientific article?. *Turk J Urol* .2013;39(Suppl 1) : 10.5152/tud.2013.046 8-9 10.5152/tud.2013.046 26328128 PMC4548565

# Response

**Published:** 2023-11-16

**Authors:** ALSAYED SHANB

**Affiliations:** Physical Therapy, Imam Abdulrahman Bin Faisal University, Dammam, Eastern Province, Saudi Arabia

**The comments of the second reviewer and** **responses of the authors** **.**

**My grateful thanks for the your valuable and important scientific comments, all of theses comments are considered.**

**Abstract:**

***Background:***

**The words physical activities were only used in the text and the words   physical performance were omitted**

**“Retain either, "physical activities" or "physical performance".**

 This statement was rephrased in all manuscript to pulmonary function, exercise capacities, physical activities.

**Avoid duplicating similar words, "pulmonary function, functional capacities, physical activities" in the same paragraph.**

 This statement was rephrased in all manuscript to pulmonary function, exercise capacities, physical activities.

***Methods:***

***Group-*** **II *:*80 male and female matched participants" - Matched? (matched non-infected with COVID-19) participants. Non-COVID-19 or ? to be added.**

 The statement was rephrased to 80 male and female matched (non-infected with COVID-19) participants.

**The Shapiro–Wilk’s test was changed to Kolmogorov–Smirnov test.**

**As the sample size is >50, Shapiro–Wilk’s test will not be appropriate to test the normality of data. Kolmogorov–Smirnov test would be more appropriate.**

 The Shapiro–Wilk’s test was changed to Kolmogorov–Smirnov test.

**Results: Add appropriate "p-value" ie, 0.05 or 0.01 or?**

 The results show significant differences in FVC & FEV _1_ of the pulmonary function ,exercise capacities and physical activity of the work &transportations between COVID-19 and matched groups (p-value = 0.001&0.001,0.001and 0.005&0.012) respectively.

**Introduction:**

 I **ntroduction should be drafted in 3 paragraphs. Please refer to this article for detailed description: Armağan (2013 ^1^ ).**

**1st Paragraph- Provide information about the general topic of the article in the light of the current literature which paves the way for the disclosure of the objective of the manuscript (what is known?).**

**2nd Paragraph – Focus on the specific subject matter, and the issue to be focused on should be dealt with, the problem should be brought forth, and fundamental references related to the topic should be discussed (what is unknown?).**

**3rd Paragraph - Finally, our recommendations for solutions should be described, in other words our aim should be communicated. When these steps are followed in that order, the reader can track the problem, and its solution from his/her own perspective under the light of current literature (what are you going to do?).**

 The introduction was rephrasing again including 4 paragraphs.  

 Its length was adjusted as I organize the introduction into 4 paragraphs.

 The first one talking about the prevalence and hazards of COVID-19 as this is new research area.

 The second and the third included the pathology of COVID-19 and its impacts on different body systems.

 The last paragraph or the fourth explains gap of knowledge and objective of the study.

 Overall, the introduction is lengthy. Generally, the introduction should be 10% to a maximum of 20% of the total manuscript length.

**Methods:**

***Sample size:***

**Change, "sigma = 13.1, and SD = 13.1" as "sigma/SD = 13.1" as both denote the same meaning.**

 It was done.

**By calculation, it was estimated to be n=73 in each group. Then, why n=7 samples were added to each group? It is unethical to utilize 14 human valuable times when the same could have arrived without including them. Please justify.**

 The sample size is considered the minimum number of participants or number of patients must be included till the results analysis and statistical outputs to be valid and reliable, so the extra numbers make our results more valid and reliable.  

 The extra or exceeding number of sample sizes do not conflict with the ethical aspects particularly our study is a cross sectional study the attendance of patients included only two times.

 The measured variables do not have any hazards for participants, also are valuable and worthful for patients and matched participants to know their results of their assessments.

***Subjects:***

**(based on external examination of body characteristics) - reference or details to be added.**

 After physical examination, inspection and analysis checking by pulmonologist to confirm their diagnosis they were infected with COVID -19, and their conditions are classified as mild and moderate not severe according to WHO severity Scale.

***Inclusion criteria:***

**Male and female patients who diagnosed with mild & moderate COVID-19 Define "mild COVID-19" and "moderate COVID-19" or add the reference for the same**.

 Definition of Mild and moderate COVID-19 was added in the paragraph of procedure of the study with also its reference.

***Exclusion criteria* *:***

**Patients with sever COVID-19 - "severe" spelling mistake.**

 It was corrected.

**Define "severe COVID-19" or add the reference for the same.**

 Was added in the paragraph of procedure of the study with also its reference.

**Procedure of the study:**

**c) Functional capacity was measured by using the 6MWT: "Each participant was asked to walk independently on flat ground" - describe the nature of the flat ground like, non-slippery, well illuminated, etc, and walk with footwear or without footwear.**

 The statement is rephrasing with addition of some explaining important words as

 Each participant was asked to walk independently with his or her comfortable footwear on a flat, well illuminated ,non-slippery ground surface  in corridor 30-meters space for 6 minutes as fast as possible without oxygen inhalation, the results were expressed in meters.

**d) Pulse oximeter - how was the pulse oximeter held - fastened to the fingers or any other part while walking as fast as possible? Add the practical issues that might help in replicating the study in the future.**

  The statement was rephrased. A wearable wrist oxygen pulse oximeter was well fastened in the index and wrist of non-dominant hand   for every participant to measure oxygen saturation during 6MWT. 

**Statistical analysis:**

**Shapiro Wilk’s test - to be replaced with Kolmogorov–Smirnov test. As the sample size is >50, Shapiro–Wilk’s test will not be appropriate to test the normality of data. Kolmogorov–Smirnov test would be more appropriate**

 Shapiro Wilk’s test was replaced with Kolmogorov–Smirnov test.

**Avoid using abnormal distribution. Use, not normal distribution.**

 It was done.

**Kruskal-Wallis’s test - When two groups (Group-I) and (Group-II), why was Kruskal-Wallis’s test used?**

 the Kruskal-Wallis’s test  was used to compare among these three groups groups matched group, Pre-6 months group and post -6 months group as the COVID -19 group was divided into two subgroups which are pre-6 months group, post -6 months group so I used the Kruskal-Wallis’s test was used to compare among three groups which are pre-6 months group, post -6 months group and matched group.

 After your suggestion and opinion and also  consultation of statistician he agreed with your opinion the sample size of matched group, pre-6 months group and post -6 months group are different, so they only compared descriptively by using confidence intervals.  and the Kruskal-Wallis’s test  was canceled.

 The Mann Whitney test was already used to compare between COVID-group & matched non-COVID-19 group. also The sample size of the pre--6 months and post -6 months sub-groups are almost similar they compared by using Mann-Whitney test.

 So finally  the Kruskal-Wallis’s test was omitted and table number 5 also was omitted.

   

**Justify. Pre 6 months and post 6 months descriptions are not clear.**

 The COVID-19 group was divided into two sub-groups which are Pre-6 months group and post -6 months group to determine time effect on the recovery of course of COVID-19 as infectious disease.

**If COVID-19 group participants were divided, then each group would not have an appropriate sample size to detect the statistical difference and statistical comments could not be made. Add more clarity**. 

 After your suggestion and opinion and also  consultation of statistician he agreed with your opinion the sample size of matched group, pre-6 months group and post -6 months group are different, so they only compared descriptively by using confidence intervals. And the Kruskal-Wallis’s test was canceled.

**Results:**

**Figure 1 - Not correct. In a cross-sectional study, how is dropout possible? Please justify.**

 As the study procedure was done by attendance of participants in two-days first day to determine the proper participants who meet inclusion criteria of the research and taking the demographic data the second day for taking different measurements, some of them are already selected and take his or her demographic data but not attend in the second day for doing measurements.

**In Table 1 to Table 5, if the data does not follow normal distribution, then it should be expressed as a median with interquartile range (IQR) or geometric mean with 95% confidence interval. Mean with SD is not appropriate. Modify accordingly.**

  The mean and standard deviation was replaced with median and IQR .

**Moreover, Table 4 and Table 5 have duplicated values which were already presented in Table 3. Avoid this duplication. Tables 3-5 could be merged.**

 It was done as the Kruskal-Wallis’s test output was cancelled and table 5 was deleted.

**Discussion:**

**Add strengths of the study.**

 Was explained and clarified in different points as rephrasing gap of knowledge, used objective assessment equipment , discussion and conclusion  

**Conclusion:**

**regarding, non-severe COVID-19 and Sakaka Aljouf. These are two key facts to be added.**

 Was done.

 Finally thanks for your worthful comments.

# Reviewer report: Reviewer response for version 2

**DOI:** 10.5256/f1000research.155029.r203744

**Published:** 2023-09-15

**Authors:** Ashraf Abdelaal Mohamed Abdelaal

**Affiliations:** 1 Department of Physiotherapy, Faculty of Applied Medical Sciences, Umm Al-Qura University, Makkah, Saudi Arabia

Authors justified the requested items.

Is the work clearly and accurately presented and does it cite the current literature?

Yes

If applicable, is the statistical analysis and its interpretation appropriate?

Partly

Are all the source data underlying the results available to ensure full reproducibility?

Yes

Is the study design appropriate and is the work technically sound?

Yes

Are the conclusions drawn adequately supported by the results?

Yes

Are sufficient details of methods and analysis provided to allow replication by others?

Yes

Reviewer Expertise:

Physiotherapy for cardiovascular/pulmonary disorders and geriatrics, cardiopulmonary rehabilitation.

I confirm that I have read this submission and believe that I have an appropriate level of expertise to confirm that it is of an acceptable scientific standard.

# Reviewer report: Reviewer response for version 1

**DOI:** 10.5256/f1000research.146511.r186229

**Published:** 2023-08-16

**Authors:** Ashraf Abdelaal Mohamed Abdelaal

**Affiliations:** 1 Department of Physiotherapy, Faculty of Applied Medical Sciences, Umm Al-Qura University, Makkah, Saudi Arabia

This cross-sectional study investigated the long-term effect of mild-moderate COVID-19 infection on the pulmonary, functional capacity and physical activities variables in patients living in Sakaka Aljouf Saudi Arabia. 160 participants of both genders were allocated to either: group-I that included 80 non-severe COVID-19 patients at least 3 months after the recovery time, and group-II that included 80 healthy matched participants.

 Evaluations of the pulmonary variables (using the spirometer), the physical capacity (using the six-minute walk test), and the physical activities variables (using the International Physical Activity Questionnaire) were done for both groups.

 Results showed significantly reduced pulmonary, functional capacity and physical activities levels (at least 3-months post-Covid-19 recovery) in patients affected with COVID-19 (group-I) compared with healthy matched participants (group-II).

**Abstract:**

Background:

- “Personal performance”: better to change it to “physical performance”.

Results:

 “The results show significant differences in…”, please provide the means and SD as well as p-values.

Conclusion:

- The sentence “whatever their severity degree of infection or history of hospitalization”: It is better to modify this sentence or remove it since the current study is limited to mild-moderately affected patients and did not provide full details regarding the history of hospitalization.
- The sentence “reflections of the current results raise awareness for physical therapists to tailor the proper rehabilitative training programs for such patients”: it is better to add “on long term-basis” at the end of the sentence.

**Introduction**:

- Abbreviations (e.g., 2MWT, FVC and so on) need to be written first in full term, then to be abbreviated after that within the text.

**Methods:**

Inclusion criteria:

- Please provide more details about the Non-sever COVID-19 as it is the main inclusion criteria (at least add “who diagnosed with mild-moderate COVID-19).

Exclusion criteria:

- It is better to remove or modulate the sentence of “All participants including non-infected individuals with COVID-19” since the study included non-infected healthy matched group.

Procedure of the study:

- Please provide the full details (version, model, manufacture) of the used instrument to evaluate the Pulmonary function.
- Please provide more clarification/details about the differences between (at least 3-months post-COVID-19 recovery), (pre 6-months), (post 6-months).

**Results:**

- Were there correlations between the severity and duration post COVID-19 infection on one hand and the evaluated variables on the other hand?
- The word “sex” is better to be replaced with “gender”.
- In table 4 and 5; please provide the numbers of participants in each group.

**Discussion:**

- Please correct the following sentence: “Results of the current study indicate that post-COVID-19 patients may experience chest abnormalities including reductions in pulmonary function, decreased functional capacity, and physical activities up to one year after recovery time”. Since the average post COVID-19 duration of this study is 7.9 months.

Is the work clearly and accurately presented and does it cite the current literature?

Yes

If applicable, is the statistical analysis and its interpretation appropriate?

Partly

Are all the source data underlying the results available to ensure full reproducibility?

Yes

Is the study design appropriate and is the work technically sound?

Yes

Are the conclusions drawn adequately supported by the results?

Yes

Are sufficient details of methods and analysis provided to allow replication by others?

Yes

Reviewer Expertise:

Physiotherapy for cardiovascular/pulmonary disorders and geriatrics, cardiopulmonary rehabilitation.

I confirm that I have read this submission and believe that I have an appropriate level of expertise to confirm that it is of an acceptable scientific standard, however I have significant reservations, as outlined above.

# Response

**Published:** 2023-08-24

**Authors:** ALSAYED SHANB

**Affiliations:** Physical Therapy, Imam Abdulrahman Bin Faisal University, Dammam, Eastern Province, Saudi Arabia

Comments of reviewer one: 

**Abstract**

**Background:**

 “Personal performance”: better to change it to “physical performance”.

**Author Response:**

 Personal performance was changed to physical performance.

**Results:**

 “The results show significant differences in…”, please provide the means and SD as well as p-values.

**Author Response:**

 The means and SD as well as p-values were added.

**Conclusion:**

 The sentence “whatever their severity degree of infection or history of hospitalization”: It is better to modify this sentence or remove it since the current study is limited to mild-moderately affected patients and did not provide full details regarding the history of hospitalization.

 The sentence “reflections of the current results raise awareness for physical therapists to tailor the proper rehabilitative training programs for such patients”: it is better to add “on long term-basis” at the end of the sentence.

**Author Response:**

 The sentence “whatever their severity degree of infection or history of hospitalization was removed.

 On long term-basis was added at the end of the sentence.

**Introduction**:

 Abbreviations (e.g., 2MWT, FVC and so on) need to be written first in full term, then to be abbreviated after that within the text.

**Author Response:**

 Abbreviations (e.g., 2MWT, FVC and so on) were done.

**Methods:**

**Inclusion criteria:** Please provide more details about the Non-sever COVID-19 as it is the main inclusion criteria (at least add “who diagnosed with mild-moderate COVID-19).

**Author Response:**

 The patient sample was selected by pulmonologist. The severity of the infection was classified according to the WHO regression scale (Marshall et al., 2020). The scale classifies the severity of COVID-19 infection into five categories: 1-uninfected with a 0 score, 2- a mild disease with a score ranging from 1-3, 3- a moderate disease with a score ranging from 4-5, 4- a severe disease with a score ranging from 6-9, and 5- dead with score 10.

**Exclusion criteria:**

 It is better to remove or modulate the sentence of “All participants including non-infected individuals with COVID-19” since the study included non-infected healthy matched group.

**Author Response:**

 Patients with COVID-19 who cannot walk, acute infections, recent surgeries.

**Procedure of the study:**

 Please provide the full details (version, model, manufacture) of the used instrument to evaluate the Pulmonary function.

**Author Response:**

 Medical international research Manufacturer’s address:

**MIR S.p.A. - Medical International Research**

 via del Maggiolino, 125

 00155 ROME (ITALY)

 Tel + 39 0622754777 Fax + 39 0622754785

 Web site: www.spirometry.com Email: mir@spirometry.com

**MIR USA, Inc.**

 5462 S. Westridge Drive

 New Berlin, WI 53151 - USA

 Tel + 1 (262) 565 – 6797 Fax + 1 (262) 364 – 2030

 Web site: www.spirometry.com Email: mirusa@spirometry.com

 https://spirometry.com/en/products/spirobank-ii-basic

 Please provide more clarification/details about the differences between (at least 3-months post-COVID-19 recovery), (pre 6-months), (post 6-months).

**Author response:**

 The covid group was selected at least 3 months from recovery after that they are classified into two groups, pre 6 months, & post 6 months after recovery time.

**Results:**

 Were there correlations between the severity and duration post COVID-19 infection on one hand and the evaluated variables on the other hand?

 The word “sex” is better to be replaced with “gender”.

 In table 4 and 5; please provide the numbers of participants in each group.

**Author Response:**

 The spearman's correlation with the severity of infection showed that positive correlation between 6 MWT physical activity transportation, and physical activity domestic &yard (r= 0.005 &0.03 respectively also presence of positive correlation between FEF _a_ & FEF _25-75_ (r= 0.042) , While the results show negative correlation BMI & physical activity transportation & leisured and free time (r= 0.015 &0.012) respectively whereas presence of positive correlation between 6MWT & FEV1 (r= 0.014).

 In addition to time factor the results show negative correlation between BMI & both physical activity of work and transportation (r= 0.015&0.027 and 0.001&0.046) in pre 6 months  and post 6 months subgroup respectively .

 The number of patients was written in the statistical part pre-6 months (30patients) and post 6-months(50 patients) sub-groups

 The word “sex” was replaced with “gender”.

**Discussion:**

 Please correct the following sentence: “Results of the current study indicate that post-COVID-19 patients may experience chest abnormalities including reductions in pulmonary function, decreased functional capacity, and physical activities up to one year after recovery time”. Since the average post COVID-19 duration of this study is 7.9 months.

**Author Response**:

 Results of the current study indicate that post-COVID-19 patients may experience chest abnormalities including reductions in pulmonary function, decreased functional capacity, and physical activities within the average time 7.9 months after recovery

**Thanks for your valuable comments.**
