# Supplementary material for: Long-term effect of non-severe COVID-19 on pulmonary function, exercise capacities and physical activities: a cross-section study in Sakaka Aljouf
Source: F1000Res. 2024 Jan 9;12:809. Originally published 2023 Jul 11. [Version 4] doi: 10.12688/f1000research.133516.4 (PMC10973945; doi:10.12688/f1000research.133516.4)
Supplement: Peer review history

# Response

**Published:** 2024-01-01

**Authors:** ALSAYED SHANB

**Affiliations:** Physical Therapy, Imam Abdulrahman Bin Faisal University, Dammam, Eastern Province, Saudi Arabia

Dear respected reviewer  Thanks for your valuable and important comments.

**First comment**

 Sample size: By calculation, it was estimated to be n=73 in each group. Then, why n=7 samples were added to each group? It is unethical to utilize 14 human valuable times when the same could have arrived without including them. Please justify.

**Response**

**1- **The sample size is considered the minimum number of participants must be included till the results analysis and statistical outputs to be valid and reliable, so the extra numbers make our results more valid and reliable.

 2-The calculated sample size   gives us an estimation of the minimum sample size,so maximizing the sample size is healthy. Sample sizes are calculated to detect the smallest clinically significant difference. to detect the smallest clinically significant difference, it has more than adequate power to detect bigger differences.   https://www.ndi.org/sites/default/files/samplesizecalculation.pdf page 17

*3-Overestimating *the same size could be done to minimize the effects of a few participants' incomplete data which is removed from analysis. 

**Second comment**

 Functional capacity was measured by using the 6MWT: "without oxygen inhalation" is not correct. It should be changed to, "without oxygen support or assisted ventilation".

**Response**

 The statement is rephrased to

  Functional capacity was measured by using the 6MWT without oxygen support.

**Third comment**

 Figure 1 – Still Not correct. In a cross-sectional study, how is dropout possible? Please remove the word, “Dropout” from, Figure 1

 In Table 1 to Table 4, if the data does not follow normal distribution, then it should be expressed as a median with interquartile range (IQR) or geometric mean with 95% confidence interval. Mean with SD is not appropriate. Modify accordingly. Even expressing in 95% confidence interval is not appropriate. Moreover, the way of representing 95% confidence interval is not correct. Table 4 is not necessary. Why have to express it in Mean with SD which is not appropriate?

**Response**

**a-The figure was updated and the word drop out was removed**

**b- the means & standard deviation in all table was replaced with median & IQR**

**c- Table 4 as you stated it was canceled and the statements were rephrased**

**d- the writing  style of confidence interval was changed**

**very thanks for your comments**

# Response

**Published:** 2023-08-24

**Authors:** ALSAYED SHANB

**Affiliations:** Physical Therapy, Imam Abdulrahman Bin Faisal University, Dammam, Eastern Province, Saudi Arabia

Comments of reviewer one: 

**Abstract**

**Background:**

 “Personal performance”: better to change it to “physical performance”.

**Author Response:**

 Personal performance was changed to physical performance.

**Results:**

 “The results show significant differences in…”, please provide the means and SD as well as p-values.

**Author Response:**

 The means and SD as well as p-values were added.

**Conclusion:**

 The sentence “whatever their severity degree of infection or history of hospitalization”: It is better to modify this sentence or remove it since the current study is limited to mild-moderately affected patients and did not provide full details regarding the history of hospitalization.

 The sentence “reflections of the current results raise awareness for physical therapists to tailor the proper rehabilitative training programs for such patients”: it is better to add “on long term-basis” at the end of the sentence.

**Author Response:**

 The sentence “whatever their severity degree of infection or history of hospitalization was removed.

 On long term-basis was added at the end of the sentence.

**Introduction**:

 Abbreviations (e.g., 2MWT, FVC and so on) need to be written first in full term, then to be abbreviated after that within the text.

**Author Response:**

 Abbreviations (e.g., 2MWT, FVC and so on) were done.

**Methods:**

**Inclusion criteria:** Please provide more details about the Non-sever COVID-19 as it is the main inclusion criteria (at least add “who diagnosed with mild-moderate COVID-19).

**Author Response:**

 The patient sample was selected by pulmonologist. The severity of the infection was classified according to the WHO regression scale (Marshall et al., 2020). The scale classifies the severity of COVID-19 infection into five categories: 1-uninfected with a 0 score, 2- a mild disease with a score ranging from 1-3, 3- a moderate disease with a score ranging from 4-5, 4- a severe disease with a score ranging from 6-9, and 5- dead with score 10.

**Exclusion criteria:**

 It is better to remove or modulate the sentence of “All participants including non-infected individuals with COVID-19” since the study included non-infected healthy matched group.

**Author Response:**

 Patients with COVID-19 who cannot walk, acute infections, recent surgeries.

**Procedure of the study:**

 Please provide the full details (version, model, manufacture) of the used instrument to evaluate the Pulmonary function.

**Author Response:**

 Medical international research Manufacturer’s address:

**MIR S.p.A. - Medical International Research**

 via del Maggiolino, 125

 00155 ROME (ITALY)

 Tel + 39 0622754777 Fax + 39 0622754785

 Web site: www.spirometry.com Email: mir@spirometry.com

**MIR USA, Inc.**

 5462 S. Westridge Drive

 New Berlin, WI 53151 - USA

 Tel + 1 (262) 565 – 6797 Fax + 1 (262) 364 – 2030

 Web site: www.spirometry.com Email: mirusa@spirometry.com

Please provide more clarification/details about the differences between (at least 3-months post-COVID-19 recovery), (pre 6-months), (post 6-months).

**Author response:**

The covid group was selected at least 3 months from recovery after that they are classified into two groups, pre 6 months, & post 6 months after recovery time.

**Results:**

Were there correlations between the severity and duration post COVID-19 infection on one hand and the evaluated variables on the other hand?

The word “sex” is better to be replaced with “gender”.

In table 4 and 5; please provide the numbers of participants in each group.

**Author Response:**

The spearman's correlation with the severity of infection showed that positive correlation between 6 MWT physical activity transportation, and physical activity domestic &yard (r= 0.005 &0.03 respectively also presence of positive correlation between FEF _a_ & FEF _25-75_ (r= 0.042) , While the results show negative correlation BMI & physical activity transportation & leisured and free time (r= 0.015 &0.012) respectively whereas presence of positive correlation between 6MWT & FEV1 (r= 0.014).

In addition to time factor the results show negative correlation between BMI & both physical activity of work and transportation (r= 0.015&0.027 and 0.001&0.046) in pre 6 months  and post 6 months subgroup respectively .

The number of patients was written in the statistical part pre-6 months (30patients) and post 6-months(50 patients) sub-groups

The word “sex” was replaced with “gender”.

**Discussion:**

Please correct the following sentence: “Results of the current study indicate that post-COVID-19 patients may experience chest abnormalities including reductions in pulmonary function, decreased functional capacity, and physical activities up to one year after recovery time”. Since the average post COVID-19 duration of this study is 7.9 months.

**Author Response**:

Results of the current study indicate that post-COVID-19 patients may experience chest abnormalities including reductions in pulmonary function, decreased functional capacity, and physical activities within the average time 7.9 months after recovery

**Thanks for your valuable comments.**
